# Corpulency

**Published:** 1890-11

**Authors:** 


### CORPULENCY.

In treating obesity the aim should be to make the consumption of the fat exceed the production. Any kind of food that will support can make fat, but certain kinds make it much more rapidly than others. It remains, therefore, fitting to regulate the diet and exercise, always keeping in mind that the change should be gradual and every thing approaching starvation should be avoided.

There are medicines which aid in reducing fat, such as liquor potassæ, bromide of ammonium, iodide of potassium, acetic acid and the haloid salts of cadmium, but most of these are very powerful stimulants to the secretory organs and should never be used unless under the direct supervision of a physician. But no medicines whatever are necessary, since with the proper diet, as given below, exercise, application of cold baths with friction, the prescribed amount of sleep and the mind actively employed during the day, the reduction of fat is certain and easy of accomplishment.

Breakfast—Lean beef, mutton, game, cold fowl, or other meat except bacon, pork or veal. Any fish except eels, catfish, salmon or fresh mackerel; eggs boiled or dropped, in an omelette, most of the yolks left out. A small piece of thin dry toast from stale bread, coffee or tea without milk or sugar.

Dinner—Broths of ordinary meats, fish of any kind, except those mentioned above, prepared plainly. Meats: Mutton, beef, game, fowl, or any kind of lean meat, with the exceptions mentioned above, the fat being carefully skimmed from the gravies. Vegetables: Beans, peas, cabbage, onions, apple sauce, tomatoes, asparagus or any vegetable except beets, oyster plant, turnips, potatoes, carrots, rice, green peas and corn. A small portion of bran bread. Dessert: Fruit cooked without sugar, cheese. For drinks: Pure water.

Supper—Cold meats from dinner, a small portion of dry toast, cheese, water-ice, plain fruit without sugar, raw tomatoes, apple sauce without sugar, the whites of dropped eggs, tea without milk or sugar.

The appetite should be satisfied, but never overloaded. Fluids of all kinds should be diminished to the lowest degree consistent with comfort. The following articles of food should be most sedulously avoided: Bread, butter, milk, sugar, potatoes—sweet and white—molasses, fat meat, Indian corn, pastry. The more nearly every one of these is absolutely cut off, the more rapid will be the reduction in weight.

Cold baths are another means or help to the desired result. A swim in cold water, a vapor bath followed by the cold douche, or thorough wash from head to foot every morning in strong brine, with friction after, will afford most satisfactory aid.

Sleep—We would not recommend more than six or seven hours, and this upon a hard bed. There should be no nap during the daytime. If the person feels drowsy active exercise should be taken, which although not indispensable, is certainly helpful. The mind should be employed, as indolence stimulates obesity.

